# Preliminary outcomes of allograft and hydroxyapatite as substitutes for autograft in anterior cervical discectomy and fusion with self-locking standalone cages

**DOI:** 10.1186/s13018-021-02257-0

**Published:** 2021-02-08

**Authors:** Changsheng Yang, Wentao Zhuo, Qingchu Li, Caiqiang Huang, Huibo Yan, Dadi Jin

**Affiliations:** Department of Orthopedics, Academy of Orthopedics of Guangdong Province, The Third Affiliated Hospital of Southern Medical University, Guangzhou, China

**Keywords:** Anterior cervical discectomy, Fusion, Bone graft, Allograft, Hydroxyapatite

## Abstract

**Purpose:**

To investigate the efficacy and safety of allograft and hydroxyapatite (HA) as substitutes for autograft in anterior cervical discectomy and fusion (ACDF).

**Methods:**

In this study, 49 patients (80 segments) treated with ACDF were included and allocated into three groups [group A, autogenous iliac bone, *n* = 18; group B, allogeneic bone, *n* = 16; group C, HA, *n* = 15]. The clinical efficacy and fusion status were compared among each group. Complications were recorded in detail, and the Bazaz classification and Voice Handicap Index-10 (VHI-10) were used to detect dysphagia and dysphonia.

**Results:**

Patients exhibited similar clinical efficacy among the groups during the final follow-up. All patients in groups A and B achieved fusion compared to only 73.3% of patients in group C. Groups A and B had similar fusion score, both of which greater than that of group C. No cage subsidence was observed in group A; however, 6.3% of patients in group B and 53.3% in group C had cage subsidence. Two patients in group A (11.1%) had persistent pain at the donor site. One patient in group B had dysphagia and dysphonia (6.3%), while one patient in group C had dysphonia (6.7%).

**Conclusion:**

In ACDF, the autogenous iliac bone was the most ideal bone graft. The allogeneic bone was an acceptable substitute but risked cage subsidence and dysphagia. HA had a much lower fusion rate and a high risk of cage subsidence. Better substitutes should be further explored for ACDF.

## Introduction

Cervical spondylopathy is one of the most common degenerative diseases. Anterior cervical discectomy and fusion (ACDF) is an effective and safe surgical treatment for this disorder. The autologous iliac bone was previously considered to be the most ideal bone graft in ACDF due to its osteogenic, osteoconductive, and osteoinductive properties.

However, autogenous bone graft possessed the disadvantages such as extra trauma and chronic pain at the donor site. Hence, exploring adequate substitutes for autogenous iliac bone remains an issue [1–5]. Commercially available substitutes for autogenous iliac bone could be divided into allograft (cancellous allografts, cortical allografts, demineralized bone matrix), xenograft, synthetic graft (calcium sulfate, calcium phosphate ceramics [hydroxyapatites, tricalcium phosphate, biphasic calcium phosphate], calcium phosphate cements, bioactive glass), and growth factor products (bone morphogenetic proteins [BMPs], platelet-rich plasma [PRP]) are widely used [6]. In spinal fusion, xenograft has been scarcely reported and BMP is usually used in combination with other bone grafts. The allogeneic bone has been widely used in clinical practice as a bone graft substitute, though its efficacy and safety remain to be verified [7]. Its most common concern is its stimulation of an immunologic response [8–11] as well as its subsequent adverse events, such as its ability to achieve fusion [12–14] and dysphagia [15]. Allogeneic bone was also associated with the risk of transmitting diseases and possessed ethical issues. Hydroxyapatite (HA) was another common bone graft substitute, which is a natural mineral found in bones, estimated to account for 50% of bone mass [16]. Due to its chemical similarity with natural bone as well as its good biocompatibility and osteoconduction [17], hydroxyapatite may serve as an ideal bone graft substitute. Several authors reported its success in spinal fusion [18–20] while some other studies held opposite opinion [21]. A prospective randomized controlled study found that 89% of HA grafts had graft fragmentation and 50% of HA grafts had obvious graft subsidence in ACDF [22]. Similarly, a prospective, matched, and controlled study had to be discontinued early due to a high rate of resorption in the Chitra-HA graft (0% fusion rate) [23].

This study directly compares the clinical outcomes and fusion status among patients with different bone grafts in ACDF to investigate the efficacy of allograft and hydroxyapatite as substitutes for autogenous iliac bone. Moreover, specific complications pertaining to treatment were also recorded to evaluate treatment safety. In this paper, the application of these three kinds of bone graft materials in ACDF surgery is directly compared with each other, which has never been seen in previous articles, if any, similar articles are rare.

## Methods

### Patients

Patients who received ACDF from January 2014 to December 2018 and had clinical and radiographical follow-up were included in this study (Table 1). The inclusion criteria were patients diagnosed with cervical spondylotic myelopathy (CSM), cervical spondylotic radiculopathy (CSR), or mixed cervical spondylosis (MCS) with radiographic findings consistent with their clinical manifestations. The exclusion criteria were acute cervical spine and spinal cord injury, severe osteoporosis, and history of previous cervical surgery. Every patient was aware of that the data (without identifying information) might be used and published for the purpose of research and signed an informed consent form at admission and this study was approved by the institutional review board in our hospital.

### Clinical evaluation

The VAS (visual analog scale) score, JOA (Japanese Orthopaedic Association), score and NDI (neck disability index) were assessed at admission and during the final follow-up. Patient satisfaction was evaluated using the Odom standard [24]: excellent, no symptoms related to cervical disease and able to perform daily activities without limitations; good, moderate symptoms related to cervical disease and able to perform daily activities without significant limitations; satisfactory, slight improvement in symptoms related to cervical disease and significant limitations in daily activities; poor, no improvement in, or aggravation of, symptoms related to cervical disease and not able to perform daily activities (Table 1).

**Table 1:** Demographic data

	Group A	Group B	Group C	***P***
**Number of samples**	18	16	15	
**Mean age (years)**	52.4 ± 14.8	56.1 ± 9.6	53.9 ± 11.1	0.663
**Male/female**	9/9	8/8	9/6	0.812
**Smoker**	0	1	0	
**Hypertention**	2	5	1	0.233
**Diabetes mellitus**	2	1	2	0.856
**Duration of clinical follow-up (months)**	45.4 ± 5.8	35.5 ± 13.0	21.1 ± 4.4	0.000
**Duration of radiological follow-up (months)**	10.2 ± 7.8	9.5 ± 5.3	7.8 ± 3.0	0.491
**Operation level**				
**Single segment**	9	4	9	
**Double segments**	8	11	4	
**Three segments**	1	1	1	
**Single segment fusion/two or three segment fusion**	9/9	4/12	9/6	0.127
**Blood loss**				
**Single segment**	67.8 ± 33.1	40.0 ± 11.5	45.6 ± 59.8	0.469
**Double segments**	83.7 ± 41.0	91.8 ± 90.1	32.5 ± 20.6	0.349
**Three segments**	100 ± 0.0	150 ± 0.0	100.0 ± 70.7	
**Total**	76.7 ± 36.1	82.5 ± 80.0	49.3 ± 54.3	0.257
**The duration of operation**				
**Single segment**	75.6 ± 11.6	101.0 ± 56.8	61.7 ± 20.2	0.220
**Double segments**	118.1 ± 19.4	94.1 ± 13.8	82.5 ± 13.2	0.002
**Three segments**	170.0 ± 0.0	125.0 ± 0.0	113.5 ± 9.2	
**Total**	99.7 ± 31.3	97.75 ± 28.9	74.1 ± 24.8	0.028
**Types**				0.544
**Cervical spondylotic myelopathy**	9	6	11	
**Cervical spondylotic radiculopathy**	7	9	4	
**Mixed cervical spondylosis**	2	1	0	
**Length of hospital stay after operation(days)**	6.2 ± 2.0	5.8 ± 1.7	5.6 ± 1.3	
**Expenses (RMB:yuan)**				
**Single segment**	47805 ± 3524	47098 ± 2426	49568 ± 9089	0.765
**Double segments**	77623 ± 4481	74215 ± 3186	79645 ± 4026	0.044
**Three segments**	120404 ± 0	11249 ± 0	109499 ± 6076	
**Total**	65091 ± 20644	69750 ± 16559	65579 ± 23456	0.773

Notes: Data shown as mean ± standard deviation

Abbreviations: *P* is the *P* value of comparison among groups A, B, and C

Dysphagia and dysphonia were investigated using the Bazaz score [25, 26] (Table 2) and Voice Handicap Index-10 (VHI-10) [26] (Table 3). Patients with scores of VHI-10 not less than 11 were diagnosed as having dysphonia.

**Table 2:** Bazaz classification [25, 26]

	Symptoms	
Severity	Liquid	Solid
None	None	None
Mild	None	Rare
Moderate	None or rare	Occasional (specific food)
Severe	None or rare	Frequent (majority)

**Table 3:** The Voice Handicap Index-10 [26]

	Response				
My voice makes it difficult for people to hear me	0	1	2	3	4
I run out of air when I talk	0	1	2	3	4
People have difficulty understanding me in a noisy room	0	1	2	3	4
The sound of my voice varies throughout the day	0	1	2	3	4
My family has difficulty hearing me when I call them throughout the house	0	1	2	3	4
I use the phone less often than I would like to	0	1	2	3	4
I’m tense when talking to others because of my voice	0	1	2	3	4
I tend to avoid groups of people because of my voice	0	1	2	3	4
People seem irritated with my voice	0	1	2	3	4
People ask, “What’s wrong with your voice?”	0	1	2	3	4
0 indicates never; 1, almost never; 2, sometimes; 3, almost always; 4, always.					

The higher the score, the more serious the dysphonia, and the cumulative score ≥ 11 was considered abnormal

### Radiographic assessment

**Fig. 1:**
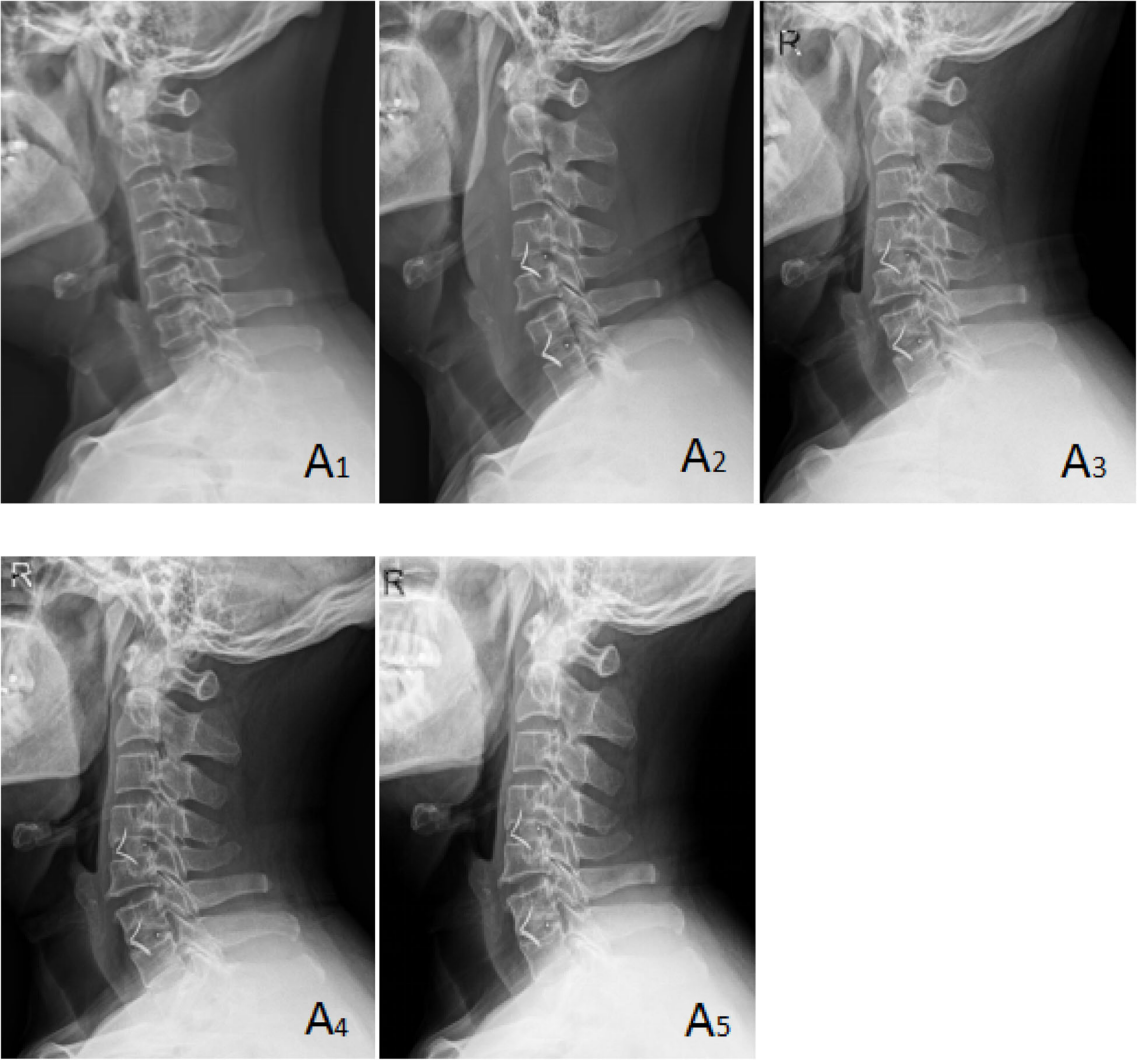
A 61-year-old male patient in group A with cervical spondylotic radiculopathy. Preoperative VAS score, JOA score, and NDI score were 7, 12, and 40%, respectively. Postoperative VAS score, JOA score, and NDI score were 1, 17, and 7%, respectively. Postoperative Odom score was “excellent.” Brantigan score: 5 points. A1 was preoperative X-ray plain film of the cervical spine, A2 was immediate X-ray plain film after operation, and A3, A4, and A5 were lateral plain film of the cervical spine at 3, 6, and 12 months after operation, respectively

**Fig. 2:**
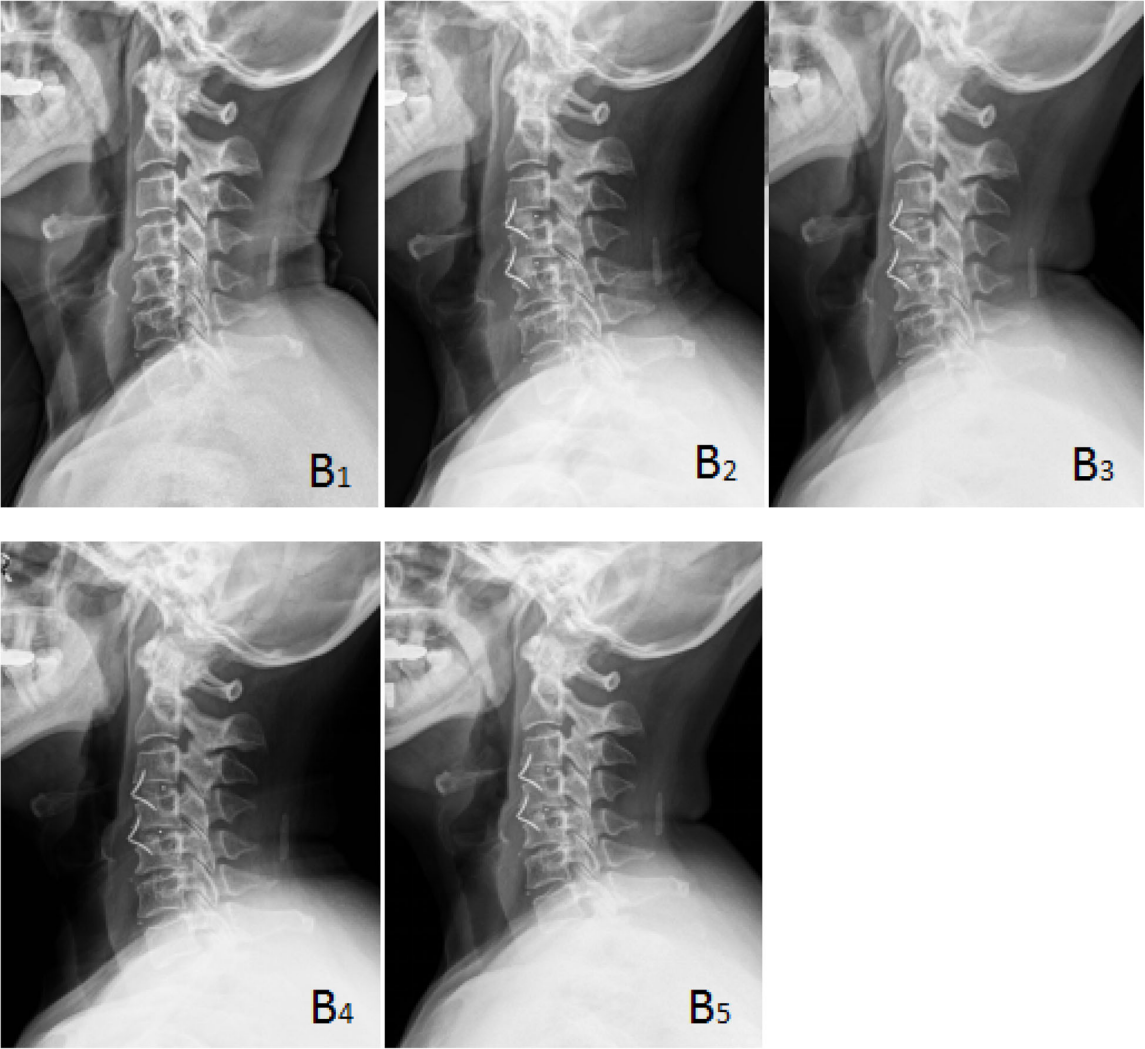
A 66-year-old female in group B with cervical spondylotic radiculopathy. The preoperative VAS score, JOA score, and NDI score were 8, 14, and 33%, respectively, and the postoperative VAS score, JOA score, and NDI score were 1, 15, and 20%, respectively. The postoperative Odom score was “excellent”. Brantigan score: 3 points. B1 was preoperative X-ray plain film of the cervical spine, B2 was immediate X-ray plain film after operation, and B3, B4, and B5 were lateral plain film of cervical spine at 3, 6, and 9 months after operation, respectively

**Fig. 3:**
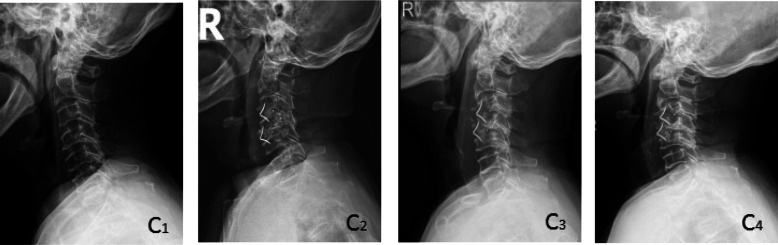
A 70-year-old female in group C with cervical spondylotic myelopathy. The preoperative VAS score, JOA score and NDI score were 5, 7, and 40% respectively, and the postoperative VAS score, JOA score and NDI score were 1, 13, and 20%, respectively. The postoperative Odom score was “excellent.” The symptoms of the patients improved obviously and there was no obvious discomfort. Brantigan score: 1, vertebral collapse, cage subsidence. C1 was preoperative X-ray plain film of the cervical spine, C2 was X-ray plain film immediately after operation, C3 and C4 were lateral plain film of the cervical spine at 3 and 6 months after operation, respectively

**Fig. 4:**
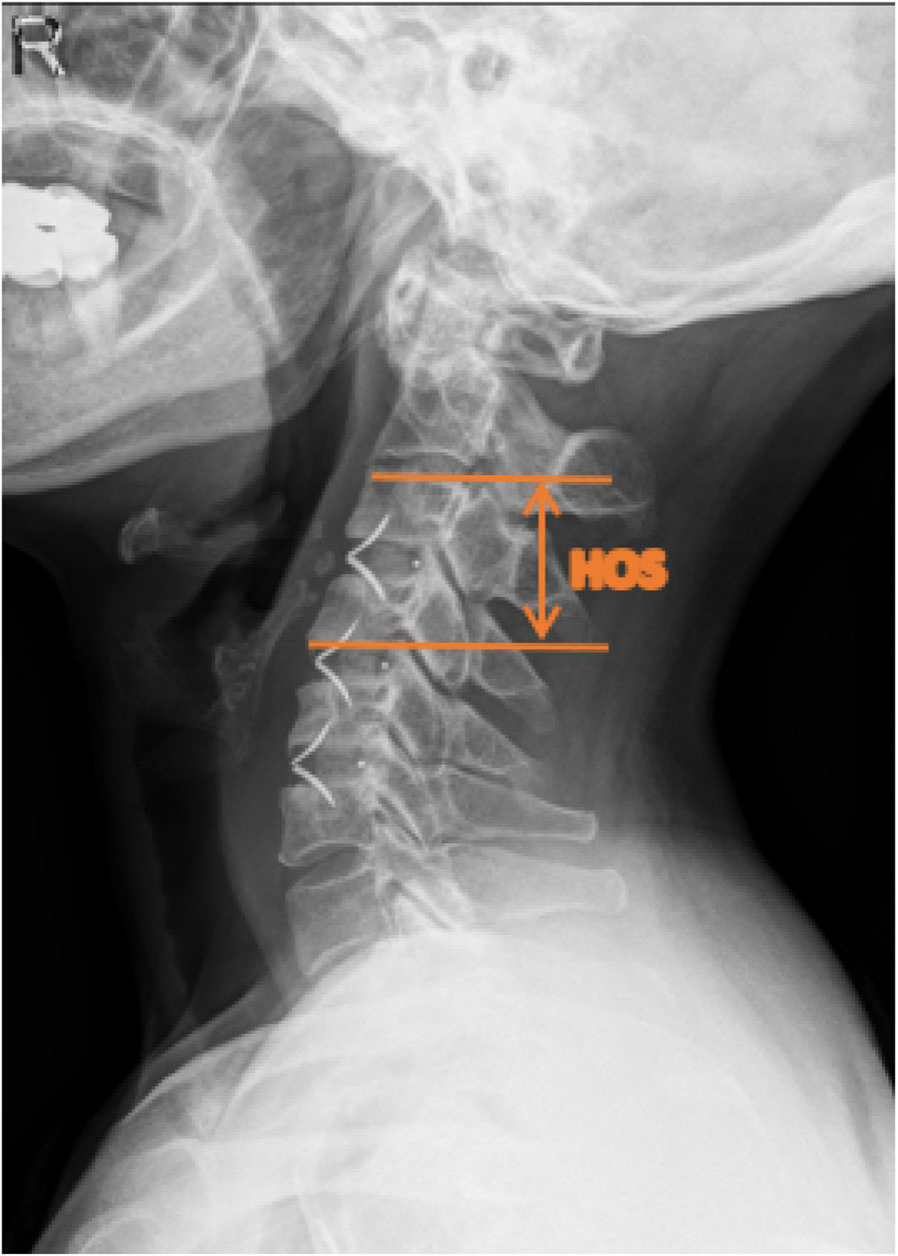
Abbreviations: HOS (height of operation segment) is the distance between the midpoint of the upper endplate of the upper vertebral body and the midpoint of the inferior endplate of the upper vertebral body on the lateral plain film of the cervical vertebra in the neutral position. For patients with double-segment or three-segment cervical surgery, the average height of the cervical spine was taken as the average

Cervical X-ray films taken in the neutral position were performed at admission, postoperatively and during the follow-up. Cage subsidence was defined as bony penetration of the implant more than 3 mm into the superior and/or inferior endplates of the adjacent vertebral body [27]. The fusion status was evaluated according to the principles (Figs. 1, 2, and 3) outlined by Brantigan et al. [28] (Table 4). The height of the operation segment (HOS) was measured on lateral X-ray films (Fig. 4). The fusion status is evaluated by Changsheng Yang and Wentao Zhuo together. Due to lack of tissue in the cervical spine, the fusion status could be clearly evaluated.

### Surgical methods

Following tracheal intubation and the administration of general anesthesia, the patient was positioned supine with the cervical spine mildly extended. An anterior cervical incision was done using the classic Robinson and Cloward anterior technique. A C-arm X-ray machine was used to locate the cervical segment of the lesion, and the corresponding intervertebral space was opened and completely decompressed. After the cartilage endplate was treated, an appropriate type of zero notch self-stability cervical fusion cage (ROI-C) was placed into the decompressed intervertebral space, fixed by double anchoring wings [29]. Patients would wear the cervical for most of time when they got up for the first 2 weeks, and they wear it only when they are outdoors in the next 4–6 weeks. The duration of operation, intraoperative blood loss, and postoperative hospital stay was recorded (Table 1).

### Statistical analysis

The preoperative VAS score, JOA score, and NDI score were respectively compared with corresponding scores during the final clinical follow-up using a paired sample *T* test. One-way ANOVA was used to compare the improvement of the VAS score, JOA score, and NDI score among the three groups during clinical follow-up, duration of operation, blood loss, HOS, and the Brantigan score. The LSD test was used in multiple comparisons, and the Welch correction and Dunnett’s T3 test were employed when there was uneven variance. The rate of clinical effect given by a remark of “excellent” or “good,” subsidence rate, and fusion rate were compared using the *R* × *C* chi-square test or the Fisher’s exact probability method. Risk factors of failed fusion were also predicted through univariable and multivariable binary logistic regression analysis. Statistical significance was set at *p*< 0.05, and the results are presented as the mean ± SD.

## Results

### Demographic data

In total, 49 patients (80 segments) treated with ACDF were included and allocated into three groups [group A, autogenous iliac bone, *n* = 18; group B, allogeneic bone, *n* = 16; group C, HA, *n* = 15]. Accordingly, no significant differences in age, gender distribution, ratio of single-segment/two or three-segment fusion and length of hospital stay after operation were found. Groups A and B had longer operation times and more blood loss than that of group C. Since HA was introduced recently, group C had a much shorter clinical follow-up time; however, radiographical follow-up time was similar among all groups (Table 4).

**Table 4:** Five-grade criteria of Brantigan et al (Brantigan score) [28]

5	Radiographic fusion	The bone in the fusion area is radiographically more denser and more mature than originally achieved in surgery. And no lucency could be detected between the graft bone and cage with vertebral bone.
4	Probable radiographic fusion	Bone bridges the entire fusion area with at least the density originally achieved at surgery, There should be no lucency between the donor bone and vertebral bone.
3	Radiographic status uncertain	Bone graft is visible in the fusion area at approximately the density originally achieved surgically. A small lucency or gap may be visible involving just a portion of the fusion area with at least half of the graft area showing no lucency between the graft bone and vertebral bone.
2	Probable radiographic pseudarthrosis	based on significant resorption of the bone graft, or a major lucency or gap visible in the fusion area (2 mm or more around the entire periphery of the graft or cage).
1	Obvious radiographic pseudarthrosis	based on collapse of the construct, loss of disc height, vertebral slip, loose anchoring wings, displacement of the carbon cage, or resorption of the bone graft.

In this study, patients with Brantigan score ≥ 3 were regarded as fusion

### Clinical results

All patients demonstrated significant improvement in terms of their VAS score, JOA score, and NDI, and no significant differences among the groups were noted. According to the Odom criteria, 77.8% of patients in group A, 50% in group B, and 66.7% in group C had a “good” or “excellent” outcome, without significant differences among the groups (Table 5).

**Table 5:** Clinical results

	Group A	Group B	Group C	***P***
**Duration of clinical follow-up (months)**	45.4 ± 5.8	35.5 ± 13.0	21.1 ± 4.4	0.000
**Pre-VAS score**	3.7 ± 3.2	4.1 ± 2.6	4.5 ± 2.7	0.721
**Post-VAS score**	1.3 ± 1.6	1.8 ± 1.9	1.8 ± 1.5	0.564
**∆VAS score (%)**	66.3 ± 32.7	30.4 ± 83.6	42.7 ± 48.0	0.311
**Pre-JOA score**	12.6 ± 2.6	12.3 ± 1.7	12.2 ± 2.9	0.900
**Post-JOA score**	14.2 ± 2.3	13.8 ± 2.2	13.9 ± 2.9	0.868
**∆JOA score (%)**	28.8 ± 57.3	22.0 ± 61.0	30.3 ± 35.9	0.898
**Pre-NDI score**	31.0 ± 13.7	33.4 ± 18.0	36.1 ± 16.0	0.651
**Post-NDI score**	13.7 ± 11.1	17.8 ± 18.1	18.5 ± 13.9	0.593
**∆NDI score (%)**	17.2 ± 16.5	15.7 ± 22.8	17.6 ± 20.2	0.958
**Odom's criteria**				
**excellent or good**	14(77.8%)	8(50.0%)	10(66.7%)	0.255
**satisfactory**	2(11.1%)	3(18.8%)	2(13.3%)	
**bad**	2(11.1%)	5(31.3%)	3(20.0%)	

Notes: Data shown as mean ± standard deviation

Pre-VAS score is the abbreviation of preoperative VAS score, and post-VAS score is the abbreviation of preoperative VAS score. Pre-JOA score, post-JOA score, pre-NDI score, and post-NDI score are similar abbreviations

∆VAS score, ∆JOA score, and ∆NDI score showed the improvement rate of VAS score, JOA score, and NDI score after operation compared with those before operation

∆VAS score = (preoperative VAS score - postoperative VAS score)/preoperative VAS score × 100%

∆JOA score = (postoperative JOA score - preoperative JOA score)/(17-preoperative JOA score) × 100%

NDI score (%) = [(total score)/(number of items completed by subjects × 5)] × 100%; ∆NDI score (%) = preoperative NDI score (%) - postoperative NDI score (%)

*P* is the *P* value of comparison among groups A, B, and C

The post-VAS score of each group was significantly better than pre-VAS score. The *P* values of groups A, B, and C were 0.001, 0.011, and 0.004, respectively

The post-JOA score of each group was significantly better than Pre-JOA score. The P values of group A, B and C were 0.001, 0.017 and 0.020, respectively

The post-NDI score of each group was significantly better than the pre-NDI score. The *P* values of groups A, B, and C were 0.000, 0.015, and 0.004, respectively

### Radiological results

Generally, no significant differences among groups in terms of HOS on admission, postoperatively or at the final radiological follow-up existed. However, 6.3% of patients in group B and 53.3% in group C suffered from cage subsidence, while cage subsidence was not evident in group A. All patients developed solid fusion in groups A and B; however, only 73.3% of patients in group C got it at the final radiological follow-up. Furthermore, the Brantigan scores in groups A and B were significantly better than that in group C (Table 6).

**Table 6:** Radiological results

	Group A	Group B	Group C	***P***
**Duration of radiological follow-up (months)**	10.2 ± 7.8	9.5 ± 5.3	7.8 ± 3.0	0.491
**HOS (mm)**				
**Preoperation**	32.2 ± 4.1	29.4 ± 4.0	30.9 ± 4.1	0.165
**Immediate-postoperation**	33.5 ± 4.0	33.6 ± 3.1	34.4 ± 4.9	0.792
**Final imaging follow-up**	33.4 ± 3.8	30.1 ± 4.5	31.9 ± 4.7	0.136
**Subsidence rate**	0.0%(0of18)	6.3%(1of16)	53.3%(8of15)	0.000
**Brantigan score**	4.6 ± 0.8	4.0 ± 0.7	3.1 ± 1.1	0.000
**Fusion rate**	100%(18of18)	100%(16of16)	73.3%(11of15)	0.000

Notes: Data shown as mean ± standard deviation

We performed univariable analyses and found that the usage of HA (OR [odds ratio] = 3.30, 95%CI [95% confidence interval] = 0.948–8.211, *p* < 0.001), female gender (OR = 2.24, 95%CI = − 0.083–7.149, *p* = 0.060), and diabetes (OR = 1.37, 95%CI = − 3.460–1.106, *p* = 0.242) were potential predictors of failed fusion. To further confirm the impact of HA on fusion, we included female gender and diabetes for adjustment in the multivariable analyses and found that usage of HA was still a predictor of failed fusion (OR = 3.13, 95%CI = 0.963–8.643, *p* = 0.007) (Table 7).

**Table 7:** Univariable and multivariable analyses to identify predictors of fusion

	Univariable analyses			Multivariable analysis		
	OR	95% CI	*P* value	OR	95% CI	*P* value
Bone graft (non-HA/HA)	3.30	0.948–8.211	< 0.001	3.13	0.963–8.643	0.007
Gender (male/female)	2.24	− 0.083–7.149	0.060	2.24	− 0.131–7.503	0.100
Diabetes (non-diabetes/diabetes)	1.37	− 3.460–1.106	0.242	0.37	− 1.432–3.764	0.791
Fused segment (single/two or three)	0.22	− 2.165–1.728	0.817			
Older age	0.40	− 1.552–2.346	0.675			

### Adverse events

In group A, one patient had chronic pain and another had hyperalgesia at the donor site. A patient with single level ACDF in group B complained of dysphagia and was only able to tolerate semi-fluid food and was diagnosed to have “serious dysphagia” according to the Bazaz classification. This patient scored 18 points according to the VHI-10 score and was evaluated to be abnormal in terms of dysphonia. One more case of dysphonia was found in group C, having 13 points in theVHI-10 score.

## Discussion

Complete decompression is an important prerequisite for the recovery of neurological function in ACDF. In this study, all operations were performed by senior spinal surgeons in our center, and all patients had significantly better VAS, JOA, and NDI scores postoperatively. Another goal in ACDF is to achieve solid fusion so as to reconstruct spinal stability and provide structural support for nerve repair. The present study investigated the efficacy of allograft and hydroxyapatite as substitutes for autograft in ACDF with fusion status as the primary outcome.

The autogenous bone had been proven effective in cervical fusion. Park et al. [30] included 32 patients with double-segmental ACDF using autogenous bone. The fusion rate was found to be 28.1%, 68.8%, 93.8%, and 93.8% at 3, 6, 12, and 24 months, respectively. In the present study, during the final radiological follow-up of group A (10.2 ± 7.8 months), all patients with autograft developed solid fusion, and no cage sinking was observed. The clinical follow-up (45.4 ± 5.8 months) of the autogenous bone group was the longest among three groups, indicating that the autogenous bone group is able to maintain long-term clinical effects. However, the autologous iliac bone required an additional surgical incision and was associated with chronic pain at the donor site. Here, two patients developed chronic pain at the donor site.

The allogeneic bone was one of the most widely used bone graft substitutes. Ryu et al. conducted a prospective study [31] and found that the imaging fusion rate of allogeneic bone at 12 months following ACDF surgery was 100% with no cage displacement. However, the allogenic bone might need more time to reach solid fusion. A prospective study performed by Suchomel et al. [32] demonstrated that the fusion rate of the autogenous bone group was 64.9% and 89.2%, while that of the allogeneic bone group was 25% and 63.1% at the 3rd and 6th month after ACDF, respectively, although the fusion rate was similar at the 12th month (94.6% versus 85.5%). Park et al. [30] found that the average duration of confirmed fusion in the allograft and autograft groups was 13.6 months and 7.7 months, respectively. In the present study, during the final imaging follow-up of group B (9.5 ± 5.3 months), the fusion rate of allograft was found to be 100%, which was the same as that of group A, but it had a lower fusion quality than that of the autogenous bone group (Brantigan score 4.0 ± 0.7 versus 4.6 ± 0.8). One case of cage sinking was found in group B.

Another problem pertaining to allogeneic bone was due to its immunogenicity, which may result in complications like dysphagia as well as reduced solid fusion. Yue et al. [15] investigated 74 patients with cervical spondylosis who had received allogeneic bone grafting before an average of 7.2 years, focusing on dysphagia associated with surgery. At the final review, 26 patients (35.1%) still suffered from dysphagia, including 12 patients with moderate dysphagia (16.2%) and 1 patient with severe dysphagia (1.4%). Miller et al. found that the incidence of dysphagia in allograft fusion was 4.3%, compared to 0% in autograft fusion. Goz et al. [33] also observed a higher prevalence in dysphagia within the allograft group. Moreover, the allogeneic bone was determined to be similar to BMP and possesses the same incidence of dysphagia. In the present study, the only dysphagia occurred in a patient treated with single-level ACDF in the allogeneic bone group. So we speculated the dysphagia in our study might be related with usage of allograft. Studies with larger sample size were warranted to confirm this observation. Other problems in relation to allografts include the difficulty of complete sterilization, spread of disease [34, 35], and ethical issues of harvest.

Another type of substitute for the autogenous bone was the artificial bone. The breakage of HA grafts was a common complication in 25% of patients in the study by Falavigna et al., although positive clinical results were observed at the same time [36]. In another prospective study, 89% of HA grafts were found to be ruptured, and 50% of HA grafts were observed to be sunken. Moreover, the further recruitment of clinical trial participants was terminated due to the high rate of graft fragmentation, and collapse was observed in subsequent radiological assessments [22]. In the present study, during the final imaging follow-up of group C (7.8 ± 3.0 months), it was found that the fusion rate of HA was 73.3%, which was significantly lower than that of patients with autograft and allograft with a decreased fusion score (3.1 ± 1.1) and high sedimentation rate (53.3%). Multivariable analyses further confirmed this observation (OR = 3.13, 95%CI = 0.963–8.643, *p* = 0.007). Thus, HA may not be applicable to ACDF. The absorption of HA is slow and could not match osteogenesis [37], and the vascularization rate of HA as an implant is also slow [38]. Other factors, such as manufacturing and porosity, may clearly influence the formation of the new blood vessels [39]; however, the present study inferred that HA should not be widely used in ACDF prior to further investigation.

### Limitations

This study had several limitations. First, it was a retrospective cohort study in a single-center with a small sample size. Second, the duration of follow-up was relatively short, although a radiological follow-up of approximately half a year is sufficient in judging bone graft fusion in patients [21]. Another limitation was that bone mineral density (BMD) data were not available because patients who were to receive ACDF would not be prescribed BMD routinely in our center. However, we compared the age and distribution of gender and found that there was no significant difference, indicating acceptable clinical heterogeneity in another profile.

## Conclusion

In ACDF, the autogenous iliac bone was the most ideal bone graft but had the disadvantage of pain in the donor site. Allogeneic bone was an acceptable substitute but had the risk of less solid fusion and dysphagia. HA had a much lower fusion rate and a high risk of cage subsidence. Better substitute remained to be explored for ACDF.

## Data Availability

All the data are included in the manuscript, and further data can be requested from the corresponding author upon reasonable request.

## Abbreviations

ACDF: Anterior cervical discectomy and fusion
HA: Hydroxyapatite
CSM: Cervical spondylotic myelopathy
CSR: Cervical spondylotic radiculopathy
MCS: Mixed cervical spondylosis
VAS: Visual analog scale
JOA: Japanese Orthopedic Association
NDI: Neck disability index
VHI-10: Voice Handicap Index-10
HOS: Height of the operation segment
